# Correction: D–π–A type conjugated indandione derivatives: ultrafast broadband nonlinear absorption responses and transient dynamics

**DOI:** 10.1039/d2ra90028a

**Published:** 2022-03-31

**Authors:** Lu Chen, Xingzhi Wu, Zhongguo Li, Ruipeng Niu, Wenfa Zhou, Kun Liu, Yingfei Sun, Zhangyang Shao, Junyi Yang, Yinglin Song

**Affiliations:** School of Physical Science and Technology, Soochow University Suzhou 215006 People’s Republic of China; School of Physical Science and Technology, Suzhou University of Science and Technology Suzhou 215009 People’s Republic of China; School of Electronic and Information Engineering, Changshu Institute of Technology Changshu 215500 China; Department of Physics, Harbin Institute of Technology Harbin 150001 People’s Republic of China

## Abstract

Correction for ‘D–π–A type conjugated indandione derivatives: ultrafast broadband nonlinear absorption responses and transient dynamics’ by Lu Chen *et al.*, *RSC Adv.*, 2022, **12**, 8624–8631, DOI: 10.1039/D2RA00349J.

The authors regret that contact details for the corresponding authors were not provided. The corrected contact details for the corresponding authors are provided below.

* Corresponding author. E-mail addresses: wuxingzhi@usts.edu.cn (Xingzhi Wu), yjy2010@suda.edu.cn (Junyi Yang), ylsong@hit.edu.cn (Yinglin Song).

The Royal Society of Chemistry apologises for these errors and any consequent inconvenience to authors and readers.

## Supplementary Material

